# Supplementary material for: Engineering Modified mRNA-Based Vaccine against Dengue Virus Using Computational and Reverse Vaccinology Approaches
Source: Int J Mol Sci. 2022 Nov 11;23(22):13911. doi: 10.3390/ijms232213911 (PMC9698390; doi:10.3390/ijms232213911)
Supplement: Supplementary file 1 [file ijms-23-13911-s001.zip › Figure S1.pdf]

Supplementary Figure S1: *Conservancy analysis of multiple sequence alignment of target proteins.*

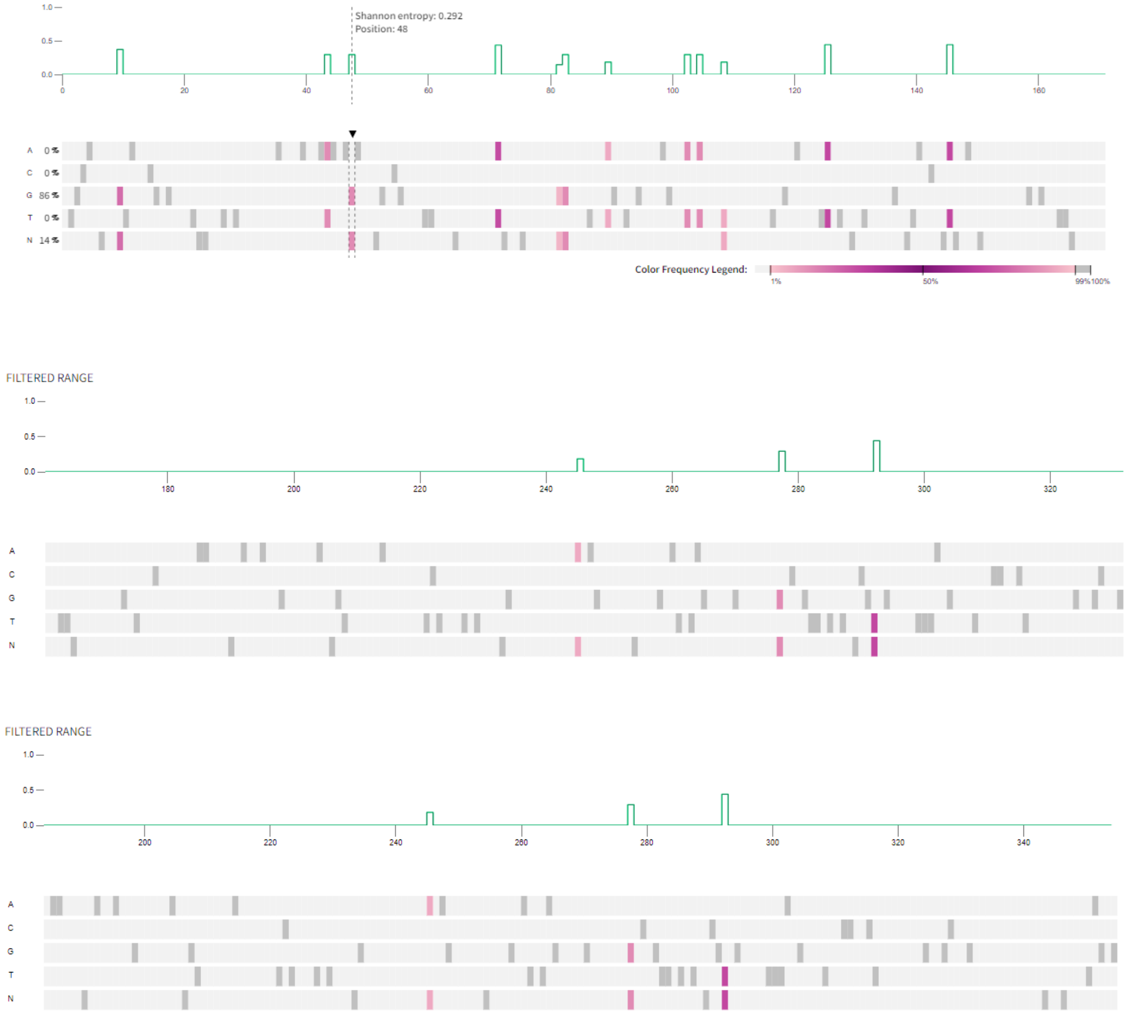

(a)

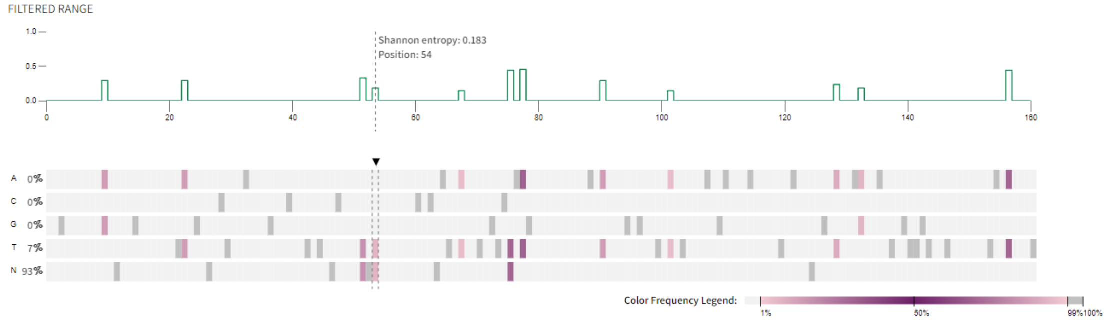

(b)

Supplementary Figure S1: *Conservancy analysis of multiple sequence alignment of target proteins.*

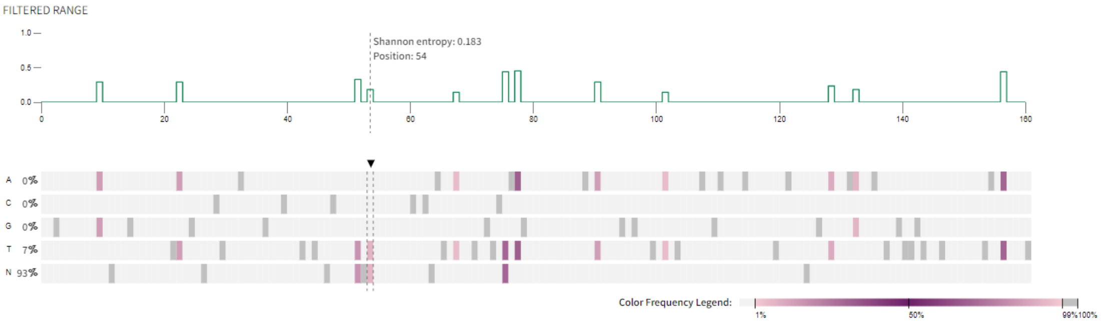

(c)

Figure S1. Conservancy analysis of multiple sequence alignment of (a) NS1 protein, (b) prM protein and (c) E protein visualized by NX4.
