# Supplementary material for: Engineering Modified mRNA-Based Vaccine against Dengue Virus Using Computational and Reverse Vaccinology Approaches
Source: Int J Mol Sci. 2022 Nov 11;23(22):13911. doi: 10.3390/ijms232213911 (PMC9698390; doi:10.3390/ijms232213911)
Supplement: Supplementary file 1 [file ijms-23-13911-s001.zip › Figure S2.pdf]

Supplementary Figure S2: *The energy dot plot for the original mRNA and for the optimized mRNA.*

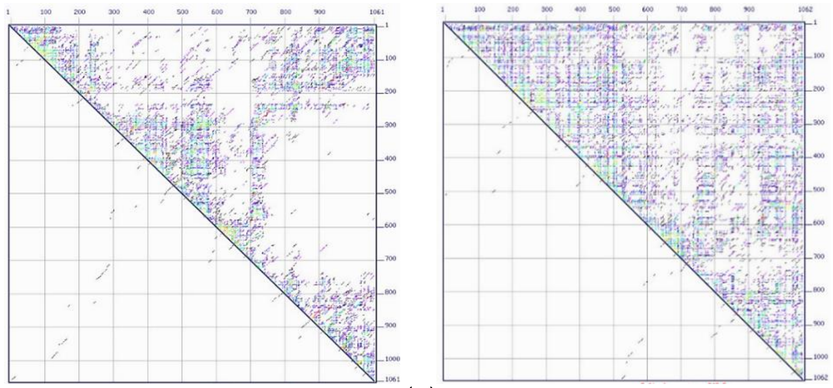

(a)

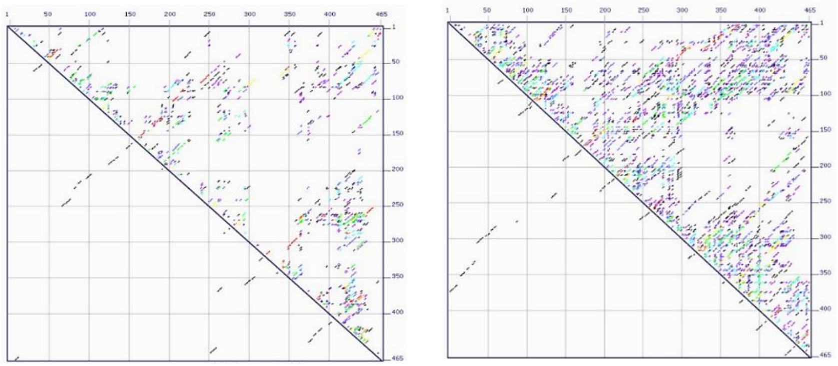

(b)

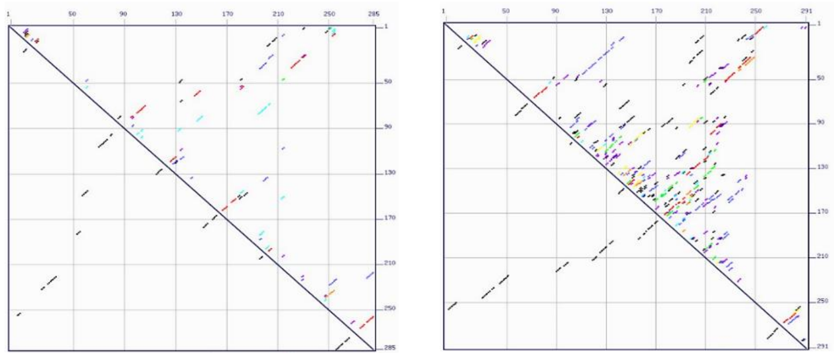

(c)

Figure S2. The energy dot plot for the original mRNA (Left) and for the optimized mRNA (Right) obtained using Mfold server. (a) The ΔG for the best calculated structure of NS1 is -418.20 kcal/mol which was higher in unoptimized structure i.e., 248.5 kcal/mol. (b) The energy for prM was -177.0 and after the optimization, ΔG for the best calculated structure was -119.2 kcal/mol. (c) The energy for EIII was 57.2 kcal/mol, which reduced to -104.4 kcal/mol after optimization.
