# Supplementary material for: Engineering Modified mRNA-Based Vaccine against Dengue Virus Using Computational and Reverse Vaccinology Approaches
Source: Int J Mol Sci. 2022 Nov 11;23(22):13911. doi: 10.3390/ijms232213911 (PMC9698390; doi:10.3390/ijms232213911)
Supplement: Supplementary file 1 [file ijms-23-13911-s001.zip › Figure S3.pdf]

Supplementary Figure S3: *Structure validation of consensus proteins (NS1, prM and EIII) based on the Ramachandran plots.*

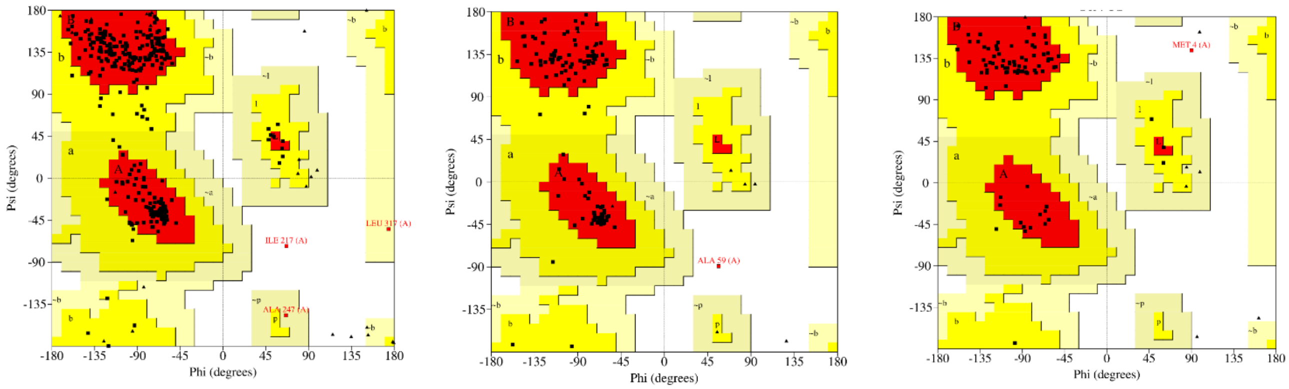

(a) (b) (c)

Figure S3. Structure validation of candidate consensus proteins based on the Ramachandran plot (a) NS1 consensus protein shows that 90.6% residues in favored region, 8.4% in allowed region and 0.3% in disallowed region; (b) Structure validation of prM consensus protein shows to have 95.7% residues in favored region, 3.6% in allowed and 0.7% in outliers region; (c) Structure validation of EIII consensus protein based on the Ramachandran plot predicts that 93.8% residues lie in favored region, 4.9% in allowed and 1.2% in outliers region.
