# Supplementary material for: Engineering Modified mRNA-Based Vaccine against Dengue Virus Using Computational and Reverse Vaccinology Approaches
Source: Int J Mol Sci. 2022 Nov 11;23(22):13911. doi: 10.3390/ijms232213911 (PMC9698390; doi:10.3390/ijms232213911)
Supplement: Supplementary file 1 [file ijms-23-13911-s001.zip › Figure S4.pdf]

Supplementary Figure S4. *Prediction of B-cell epitope of EIII protein of dengue virus.*

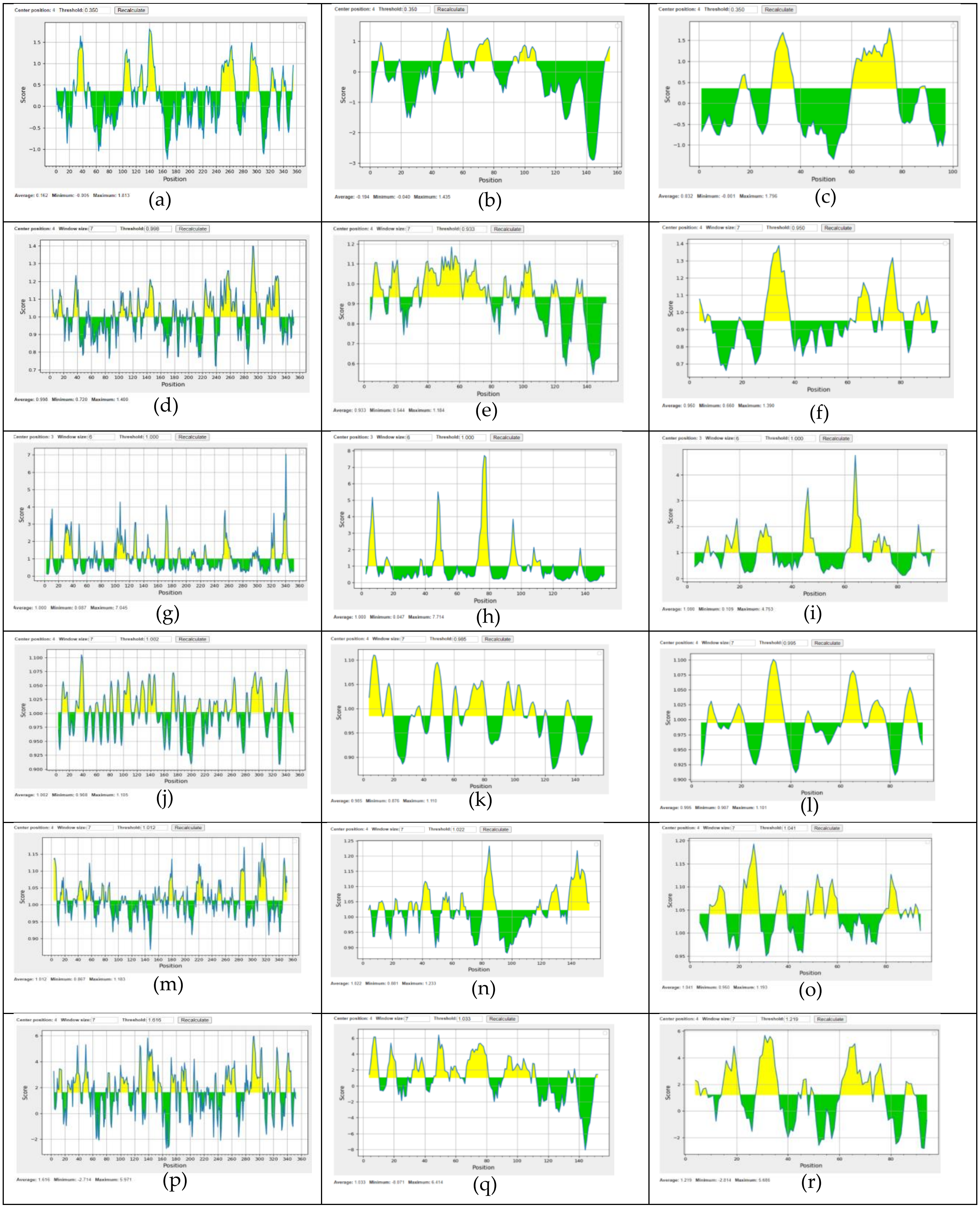

Supplementary Figure S4. *Prediction of B-cell epitope of EIII protein of dengue virus.*

Figure S4. Prediction of B-cell epitope of EIII protein of dengue virus. The prediction methods include: BepiPred linear epitope prediction (a) NS1, (b) prM (c) EIII; Chou–Fasman beta turn prediction (d) NS1, (e) prM, (f) EIII; Emini Surface accessibility prediction (g) NS1, (h) prM, (i) EIII; Karplus and Schulz flexibility prediction (j) NS1, (k) prM, (l) EIII; Kolaskar and Tongaonkar antigenicity (m) NS1, (n) prM, (o) EIII; Parker hydrophilicity prediction (p) NS1, (q) prM, (r) EIII.
