# Supplementary material for: Engineering Modified mRNA-Based Vaccine against Dengue Virus Using Computational and Reverse Vaccinology Approaches
Source: Int J Mol Sci. 2022 Nov 11;23(22):13911. doi: 10.3390/ijms232213911 (PMC9698390; doi:10.3390/ijms232213911)
Supplement: Supplementary file 1 [file ijms-23-13911-s001.zip › Figure S5.pdf]

***Supplementary Figure S5.*** *The 3D structure of the selected T-cell epitopes.*

| Proteins | Epitopes (MHC-I binders) | | | Epitopes (MHC-II binders) |
|---|---|---|---|---|
| **NS1** | 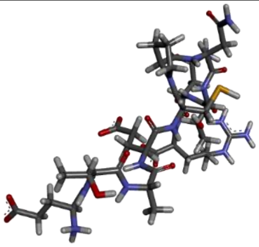 ETAECPNTNR | 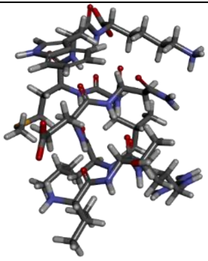 VTRLENLMWK | 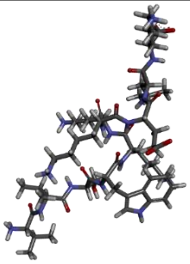 VVSWKKKELK | 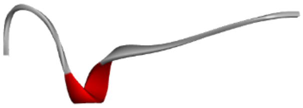 VFTTNIWLKLKERQ |
| **prM** | 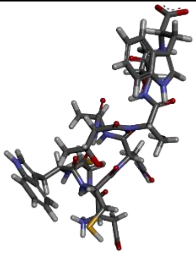 MGLETRTETW | 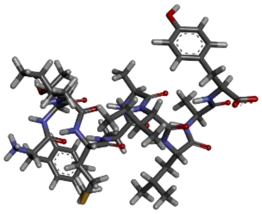 FTIMAAILAY | 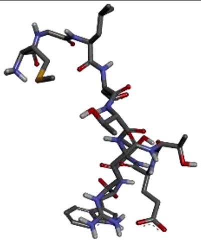 DCWCNATSTW | 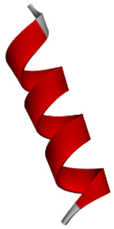 RHPGFTIMAAILAYT |
| **EIII** | 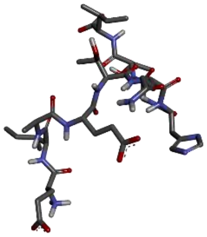 EIAETQHGTI | 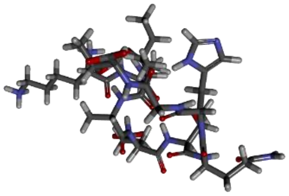 KEIAETQHGT | | 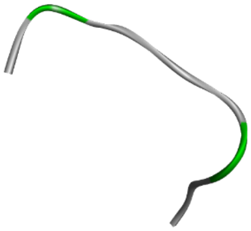 KIPFEIMDLEKRHVL |

**Figure S5.** The 3D structure of the selected T-cell epitopes was generated by the trRosetta peptide structure prediction server.
