# Supplementary material for: Engineering Modified mRNA-Based Vaccine against Dengue Virus Using Computational and Reverse Vaccinology Approaches
Source: Int J Mol Sci. 2022 Nov 11;23(22):13911. doi: 10.3390/ijms232213911 (PMC9698390; doi:10.3390/ijms232213911)
Supplement: Supplementary file 1 [file ijms-23-13911-s001.zip › Figure S6.pdf]

Supplementary Figure S6: *Docking and favorable interactions between epitopes and their corresponding alleles.*

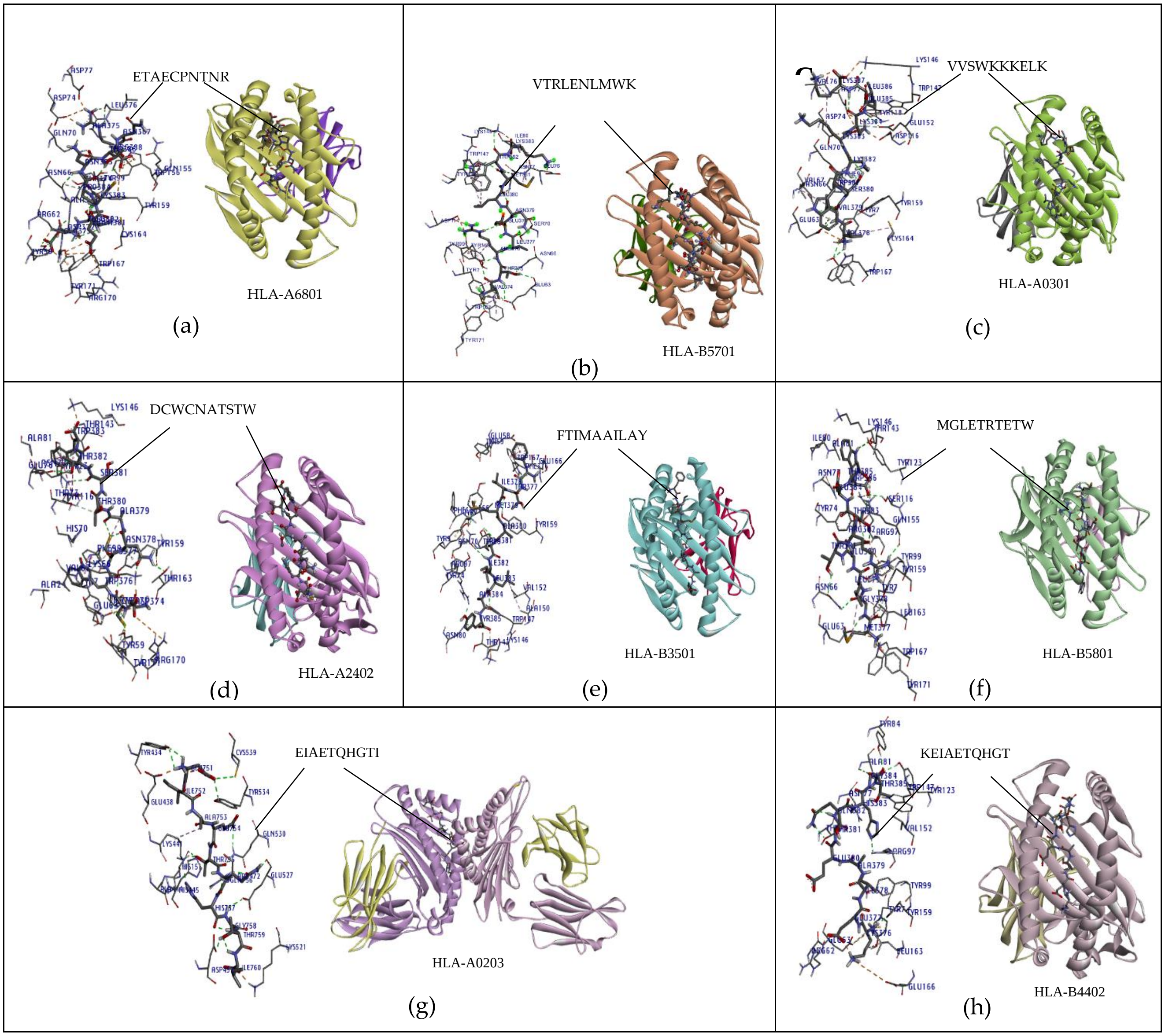

Figure S6. The interactions of MHC-I epitopes from NS1 protein with their corresponding alleles. **(a)** The epitope ETAECPNTNR binds with HLA-A*68:01 with six favorable interactions; **(b)** The epitope VTRLENLMWK showed 9 favorable interactions with HLA-B*57:01; **(c)** The epitope VVSWKKKELK showed 7 favorable interactions with HLA-A*03:01; **(d)** Epitopes DCWCNATSTW of prM were able make 8 favorable interactions with their corresponding alleles HLA-A*24:02; **(e)** The epitope FTIMAAILAY of prM made 8 favorable interactions with their corresponding allele HLA-B*53:01; **(f)** The epitope MGLETRTETW of prM made seven favorable interactions with HLA-B*58:01; **(g)** The epitope EIAETQHGTI of EIII formed 5 favorable interactions with HLA-A*02:03; **(h)** Seven favorable interactions were observed between KEIAETQHGT of EIII and HLA-B*44:02.
