# Supplementary material for: Engineering Modified mRNA-Based Vaccine against Dengue Virus Using Computational and Reverse Vaccinology Approaches
Source: Int J Mol Sci. 2022 Nov 11;23(22):13911. doi: 10.3390/ijms232213911 (PMC9698390; doi:10.3390/ijms232213911)
Supplement: Supplementary file 1 [file ijms-23-13911-s001.zip › Figure S7.pdf]

Supplementary Figure S7: *Docking simulations between the consensus protein and Dengue receptors.*

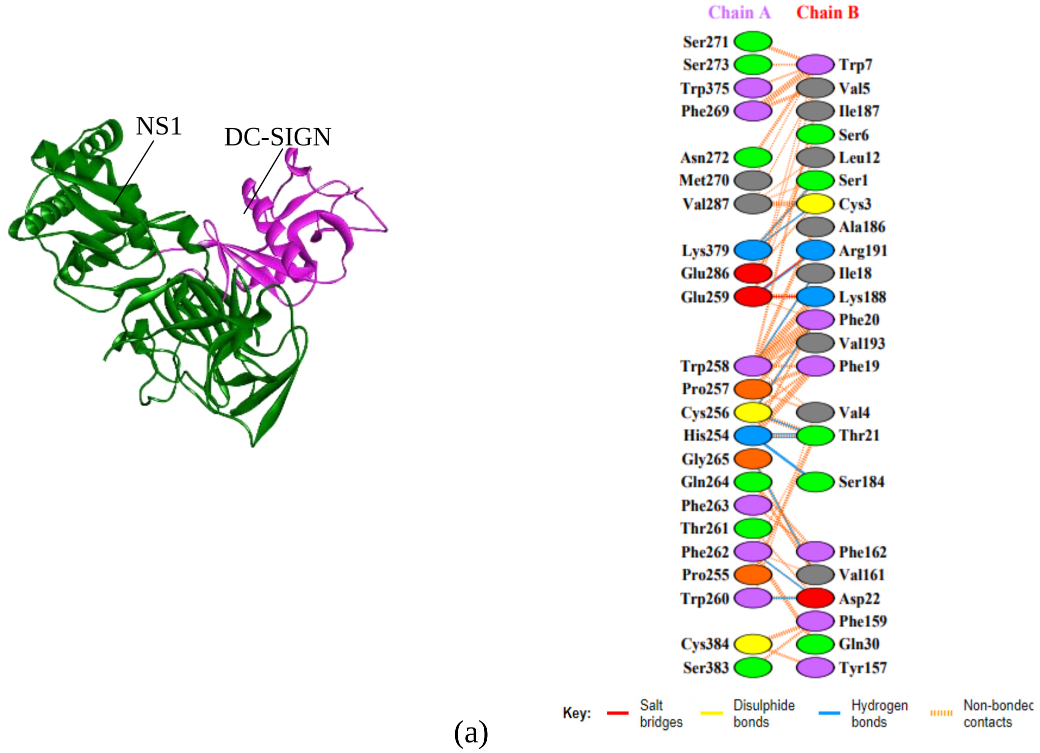

(a)

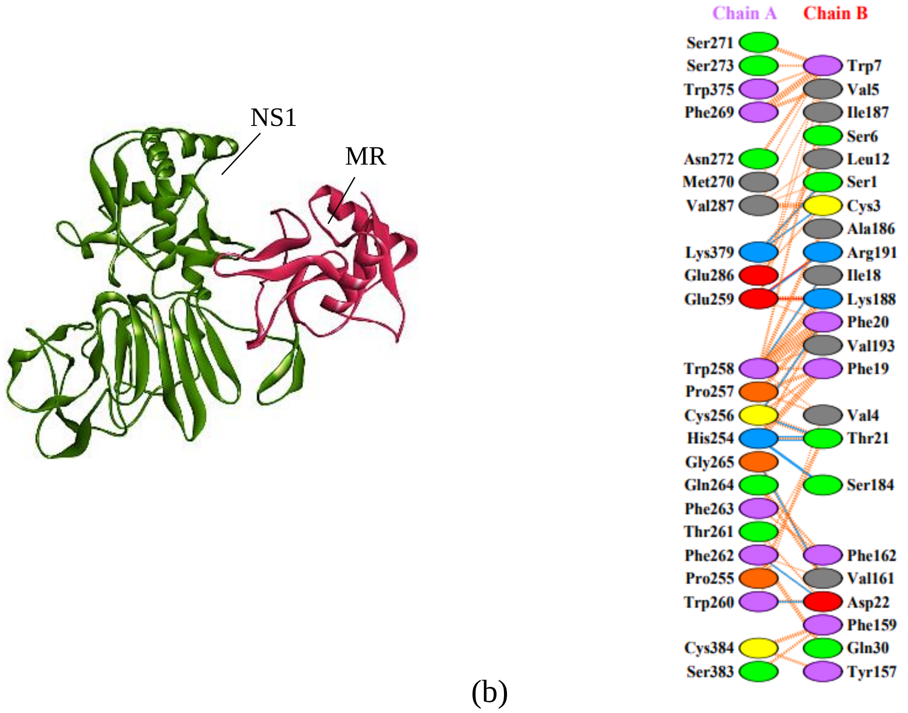

(b)

Supplementary Figure S7: *Docking simulations between the consensus protein and Dengue receptors.*

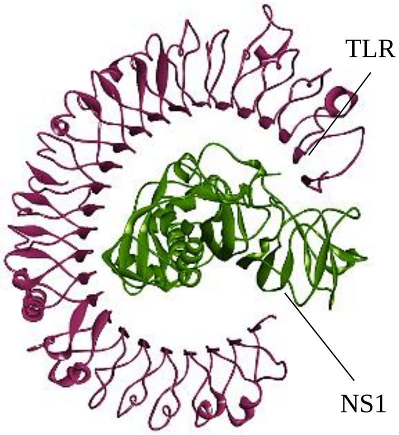

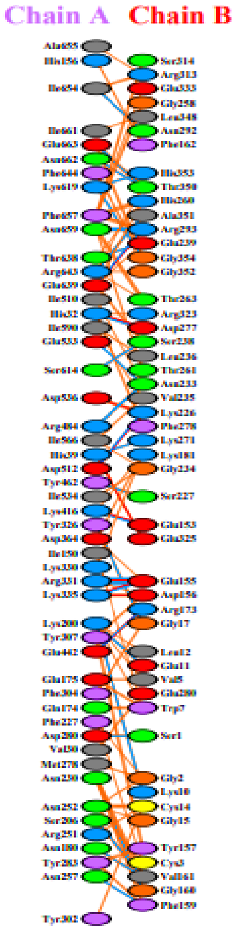

(c)

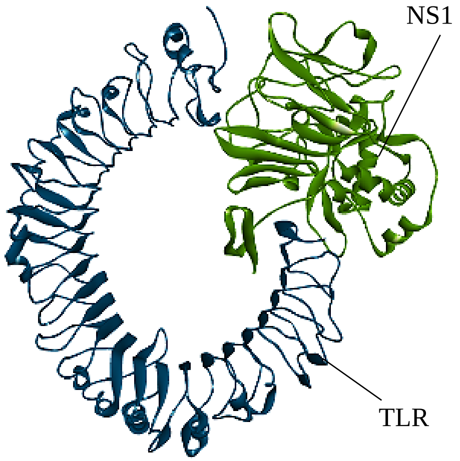

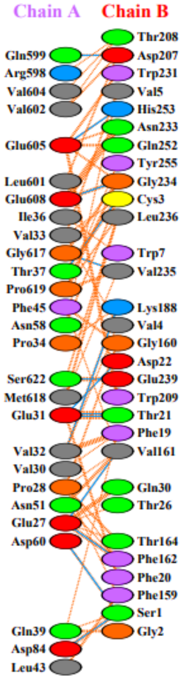

(d)

Supplementary Figure S7: *Docking simulations between the consensus protein and Dengue receptors.*

Figure S7 (i) Docking complex of NS1 protein and immune receptors (Left) and PDBsum results showing the interface residues (Right). **(a)** NS1 protein and DC-SIGN PDBsum results showed that there were 23 interface residues between, 2 salt bridges, 13 hydrogen bonds and 200 non bonded contacts **(b)** The number of interface residues between NS1 protein and MR receptor were 26 and 23 respectively with 10 hydrogen bonds and 160 non bonded contacts between them **(c)** Between NS1 protein and TLR3 receptor, 50 interface residues were observed with 10 salt bridges, 23 hydrogen bonds and 284 non bonded interactions **(d)** The interactions between 27 interface residues of TLR4 receptor with 30 interface residues of NS1 protein was supported by 14 hydrogen bonds and 158 non bonded contacts.

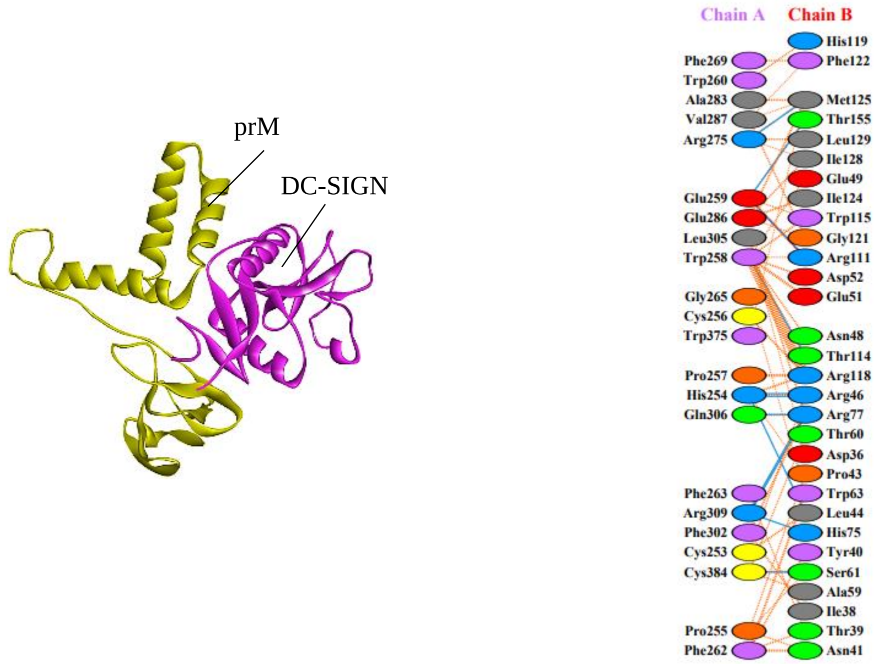

(a)

Supplementary Figure S7: *Docking simulations between the consensus protein and Dengue receptors.*

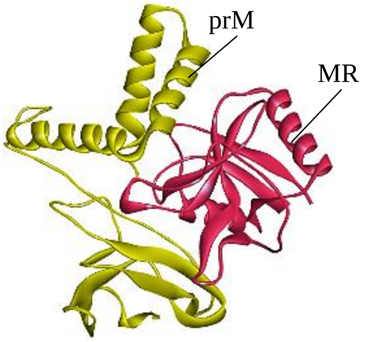

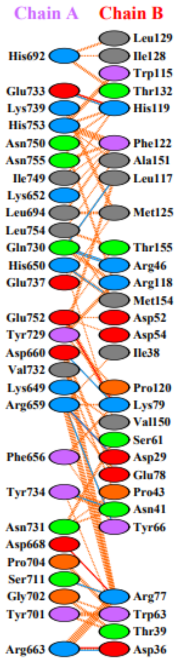

(b)

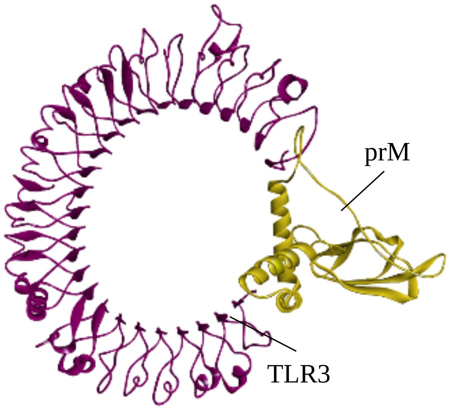

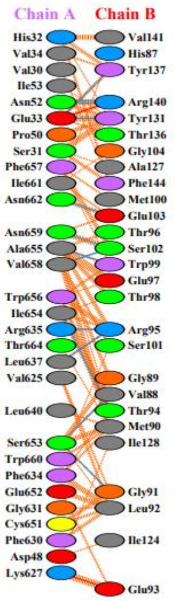

(c)

Supplementary Figure S7: *Docking simulations between the consensus protein and Dengue receptors.*

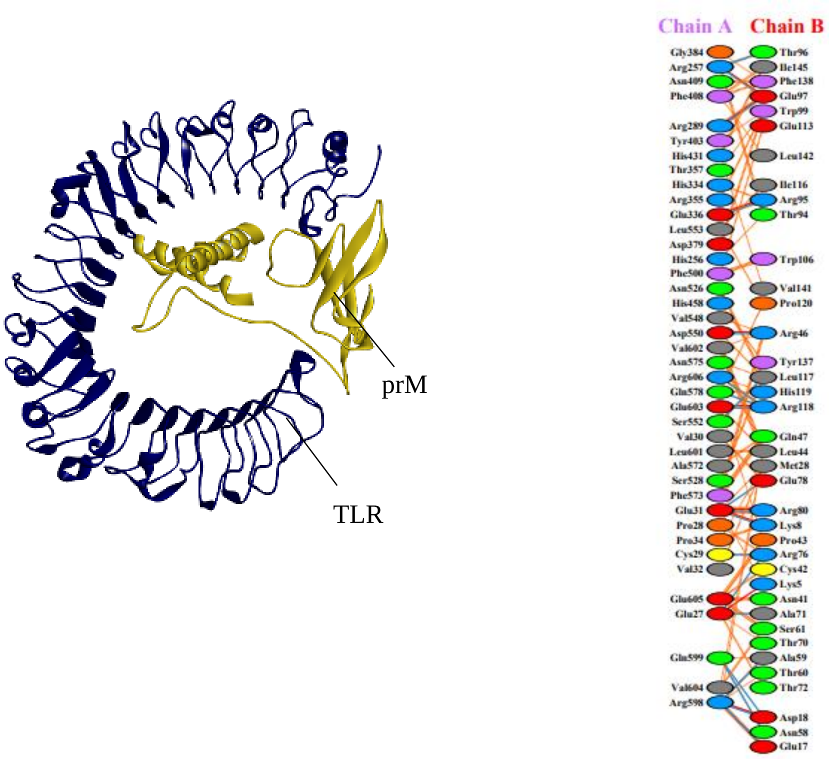

(d)

Figure S7 (ii) Docking complex of prM protein and immune receptors (Left) and PDBsum results showing the interface residues (Right). **(a)** 22 interface residues of DC-SIGN and 30 residues of prM protein interacted using 1 salt bridge, 12 hydrogen bonds and 194 non bonded contacts between them. **(b)** The number of interface residues between MR receptor and prM protein were 28 and 29 respectively with 5 salt bridges, 11 hydrogen bonds and 207 non bonded contacts **(c)** The number of interface residues observed between TLR3 and prM protein and TLR3 were 30 and 27, respectively and there were 2 salt bridges, 13 hydrogen bonds and 242 non bonded interactions **(d)** The interactions between 40 interface residues of TLR4 receptor and 38 interface residues of prM protein was supported by 10 salt bridges, 20 hydrogen bonds and 273 non bonded interactions.

Supplementary Figure S7: *Docking simulations between the consensus protein and Dengue receptors.*

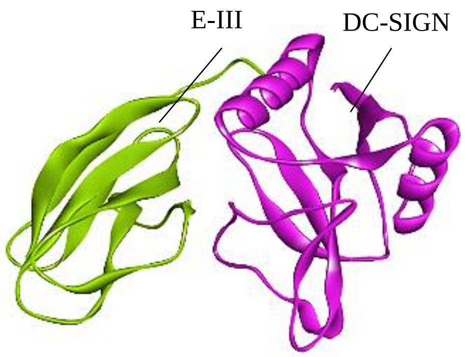

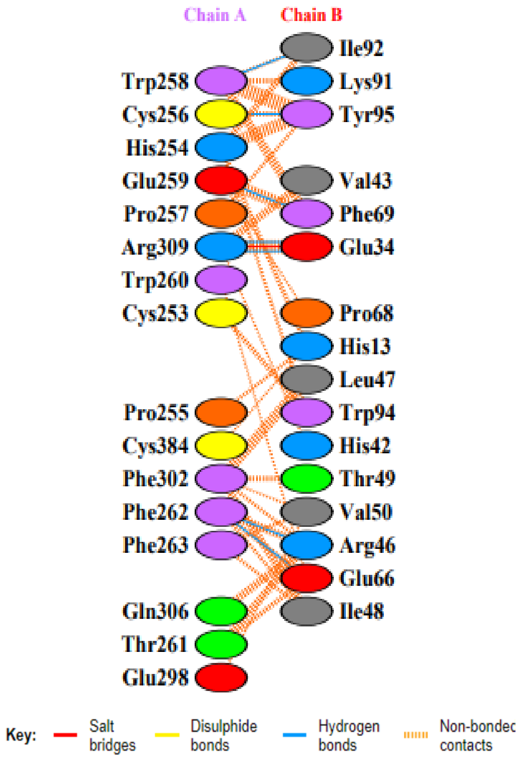

(a)

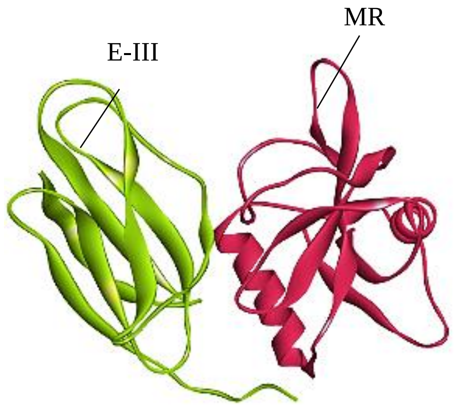

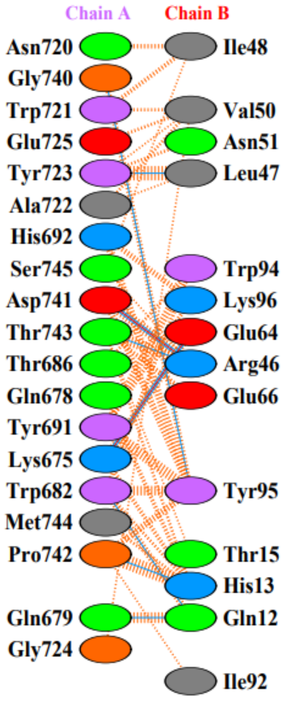

(b)

Supplementary Figure S7: *Docking simulations between the consensus protein and Dengue receptors.*

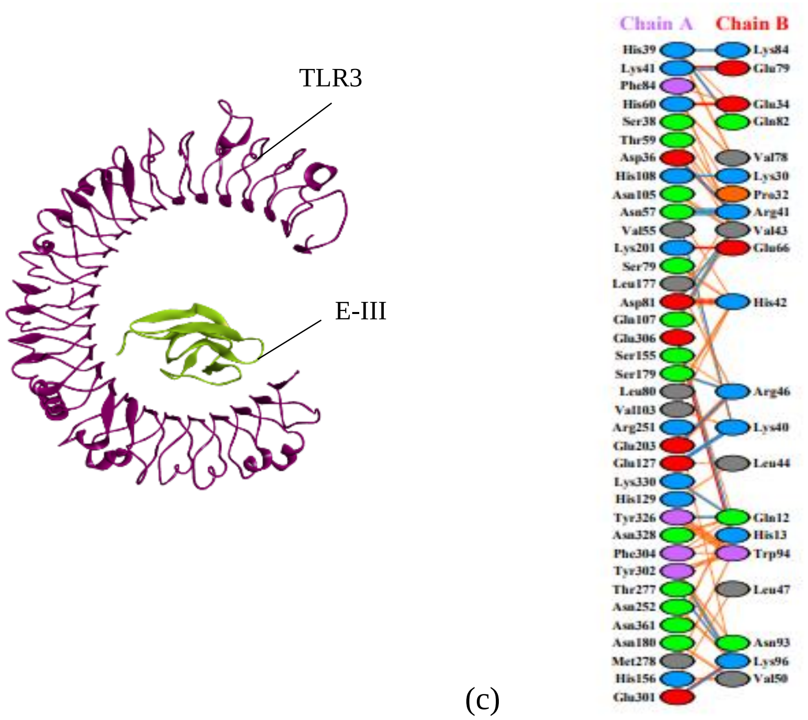

(c)

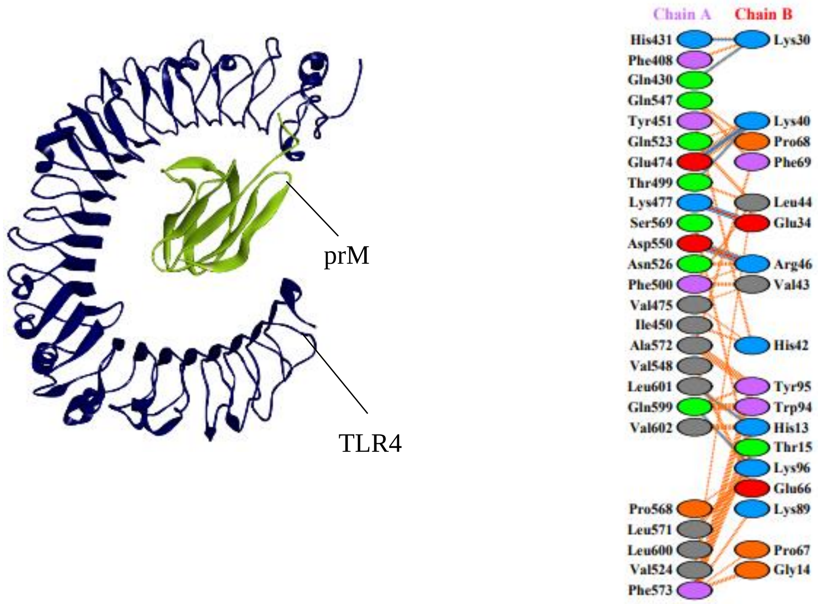

(d)

Figure S7 (iii) Docking complex of EIII protein and immune receptors (Left) and PDBsum results showing the interface residues (Right). **(a)** DC-SIGN and EIII protein interacted with eachother with 16 interface residues and 1 salt bridge, 7 hydrogen bonds and 120 non bonded contacts were

Supplementary Figure S7: *Docking simulations between the consensus protein and Dengue receptors.*

observed between them **(b)** EIII and MR receptors showed 14 and 19 interacting interface residues and 2 salt bridges, 10 hydrogen bonds and 146 non bonded contacts were present between them. **(c)** TLR3 had 37 interface residues interacting with 21 interface residues of EIII and they formed 9 salt bridges, 21 hydrogen bonds and 217 non-bonded contacts between them. **(d)** The number of interface residues between TLR4 and EIII were 25 and 18, respectively and 3 salt bridges, 11 hydrogen bonds and 125 non bonded atoms were found between them.
