# Supplementary material for: Engineering Modified mRNA-Based Vaccine against Dengue Virus Using Computational and Reverse Vaccinology Approaches
Source: Int J Mol Sci. 2022 Nov 11;23(22):13911. doi: 10.3390/ijms232213911 (PMC9698390; doi:10.3390/ijms232213911)
Supplement: Supplementary file 1 [file ijms-23-13911-s001.zip › Figure S8.pdf]

Supplementary Figure S8: *Normal Mode Analysis of Selected vaccine proteins and Dengue immune receptors.*

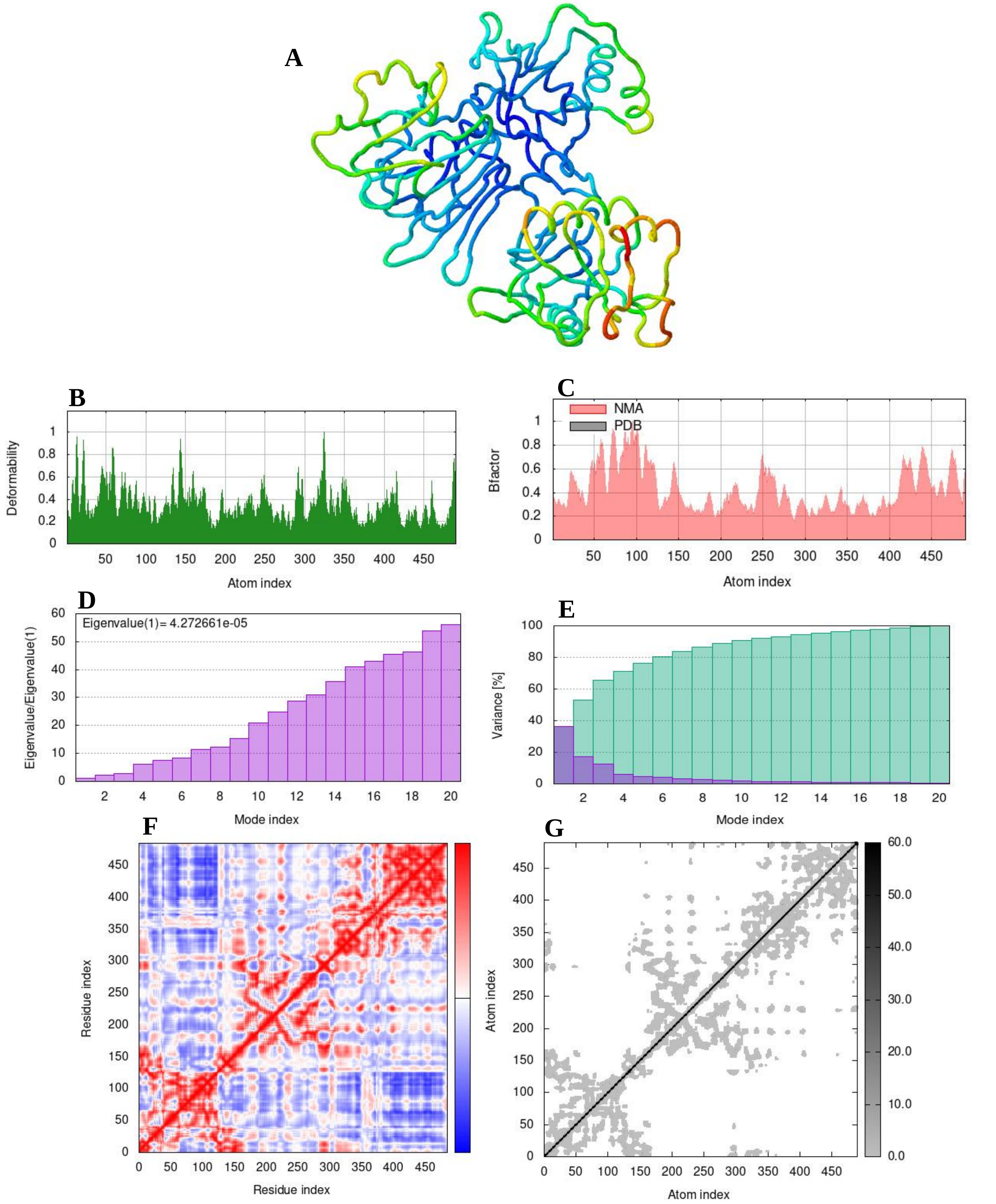

Figure S8 (i): Molecular dynamics simulation of NS1 and DC-SIGN docking complex by iMODS server. (A) NS1 and MR docking complex. (B) Main-chain deformability. (C) B-factor values. (D) The eigenvalue. (E) Variance. (F) Co-variance map. (G) Elastic network of model.

Supplementary Figure S8: *Normal Mode Analysis of Selected vaccine proteins and Dengue immune receptors.*

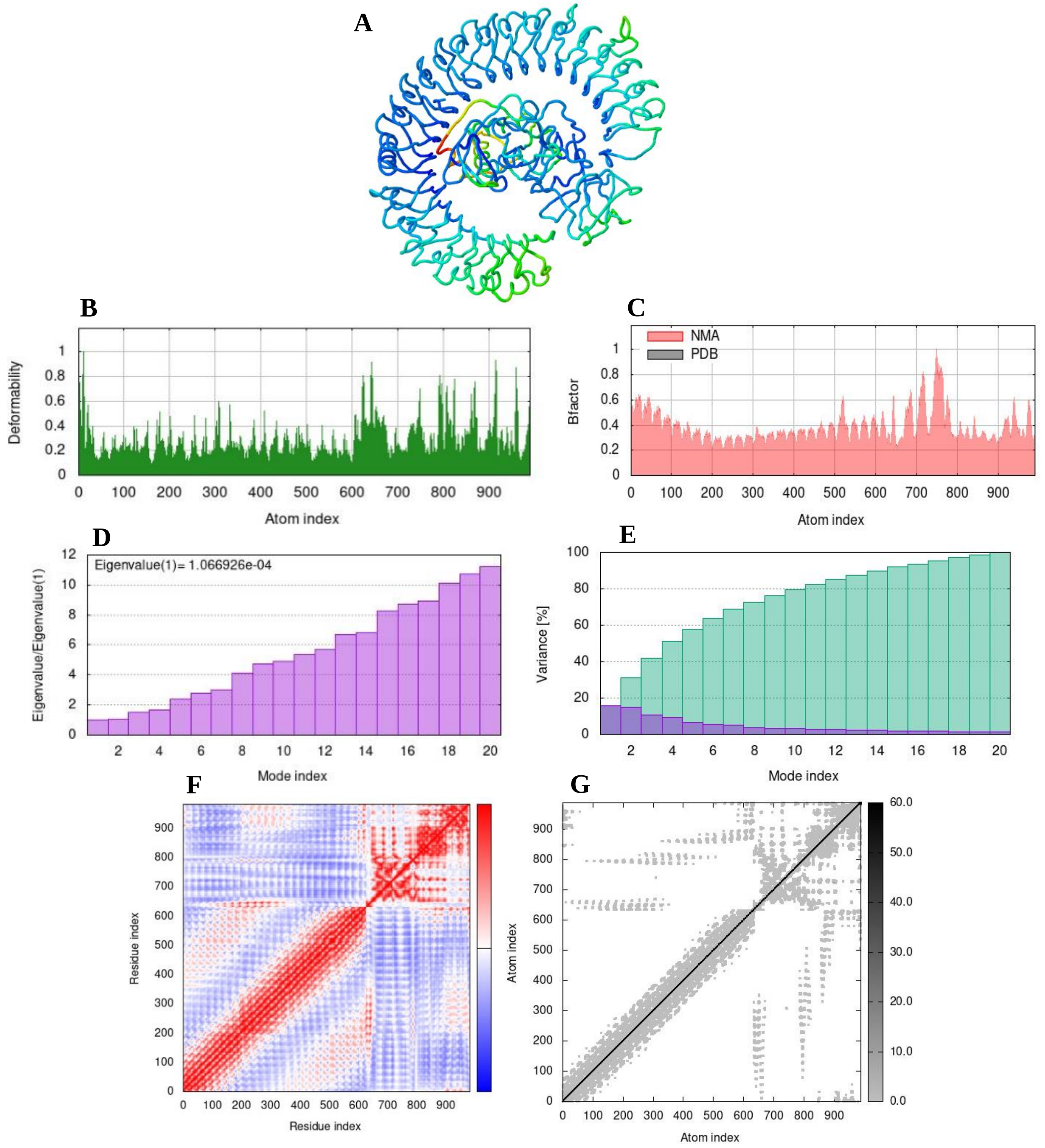

Figure S8 (ii): Molecular dynamics simulation of NS1 and TLR3 docking complex by iMODS server. (A) NS1 and TLR3 docking complex. (B) Main-chain deformability. (C) B-factor values. (D) The eigenvalue. (E) Variance. (F) Co-variance map. (G) Elastic network of model.

Supplementary Figure S8: *Normal Mode Analysis of Selected vaccine proteins and Dengue immune receptors.*

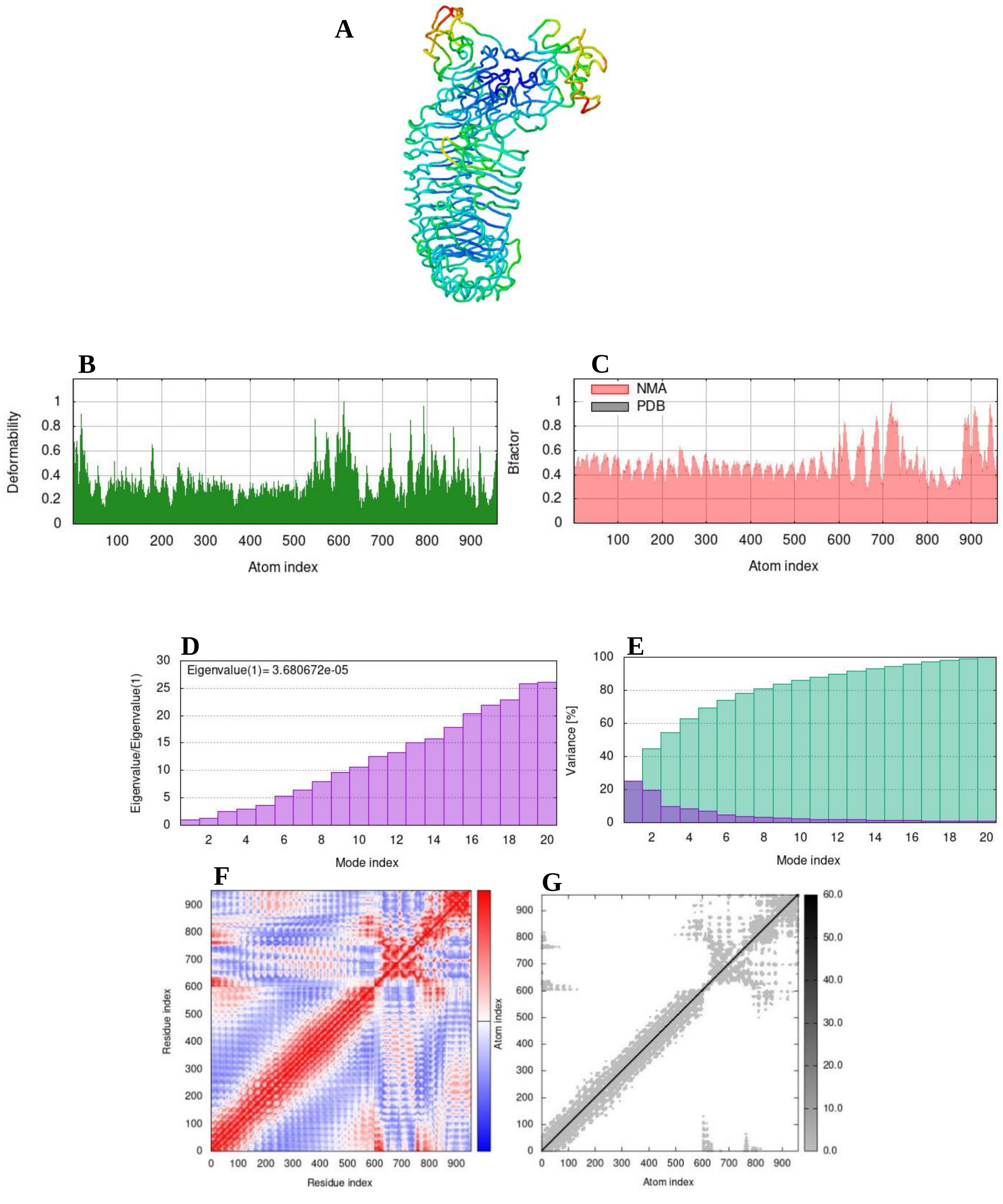

Figure S8 (iii): Molecular dynamics simulation of NS1 and TLR4 docking complex by iMODS server. (A) NS1 and TLR4 docking complex. (B) Main-chain deformability. (C) B-factor values. (D) The eigenvalue. (E) Variance. (F) Co-variance map. (G) Elastic network of model.

Supplementary Figure S8: *Normal Mode Analysis of Selected vaccine proteins and Dengue immune receptors.*

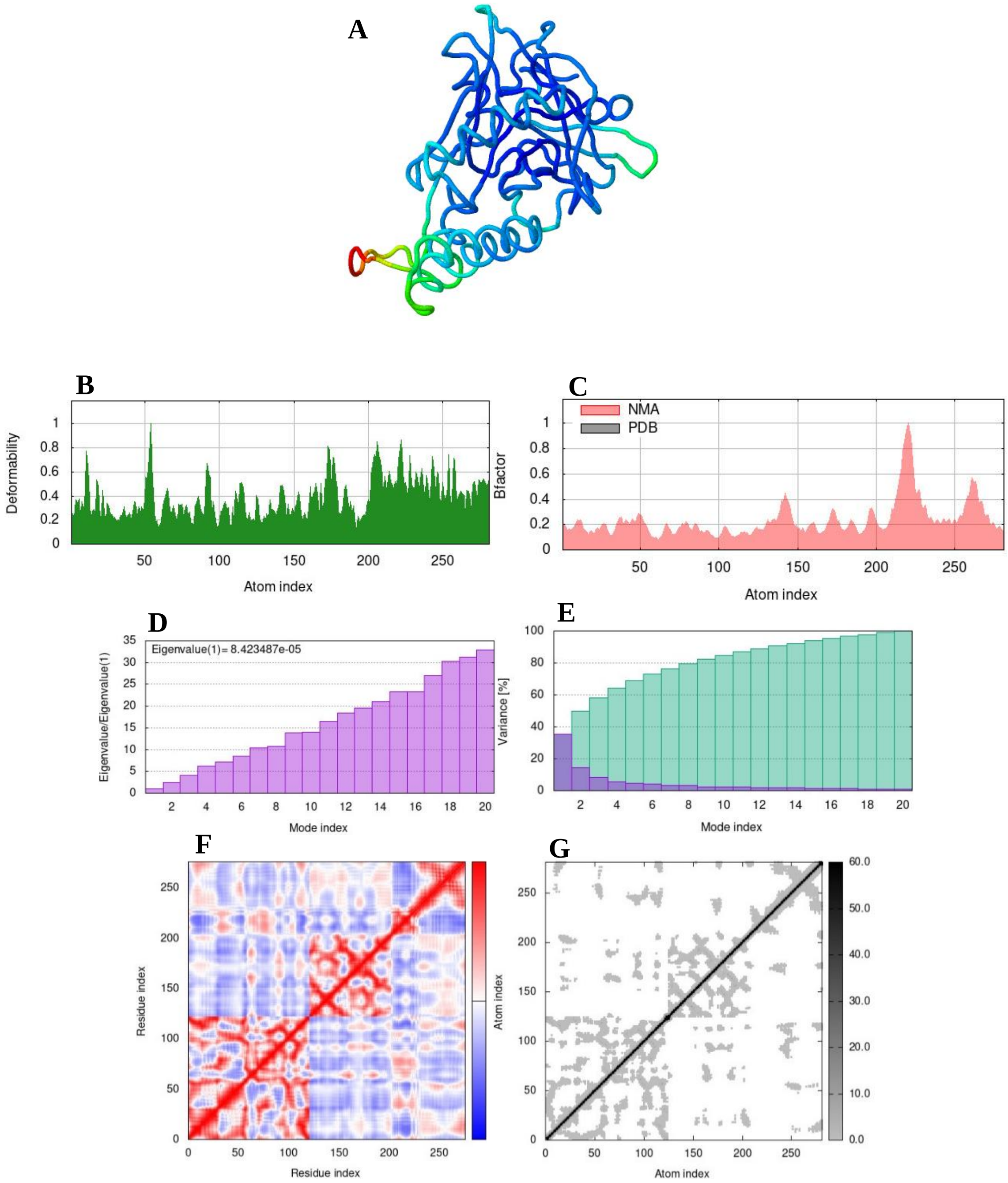

Figure S8 (iv): Molecular dynamics simulation of prM and MR docking complex by iMODS server. (A) prM and MR docking complex. (B) Main-chain deformability. (C) B-factor values. (D) The eigenvalue. (E) Variance. (F) Co-variance map. (G) Elastic network of model.

Supplementary Figure S8: *Normal Mode Analysis of Selected vaccine proteins and Dengue immune receptors.*

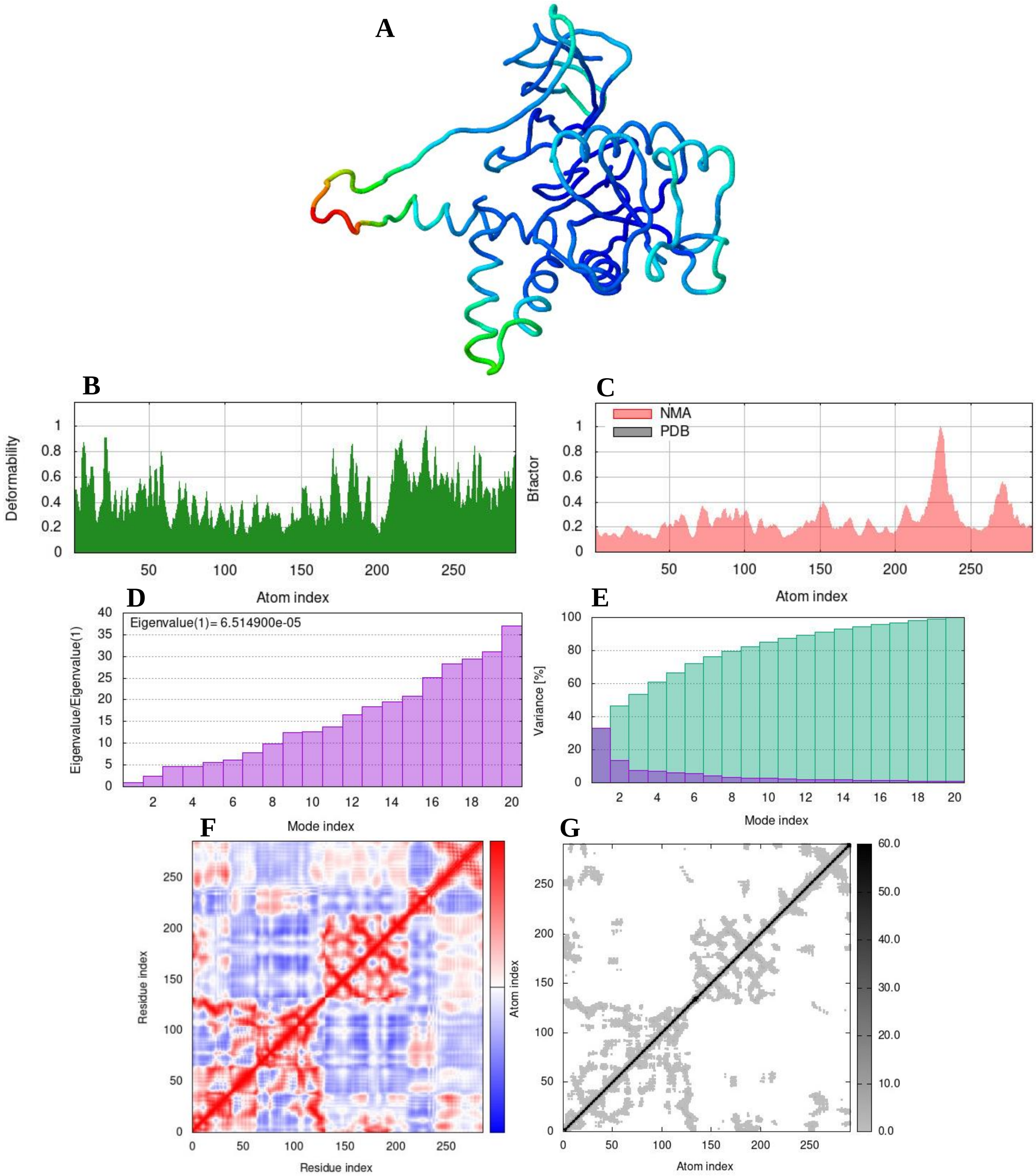

Figure S8 (v): Molecular dynamics simulation of prM and DC-SIGN docking complex by iMODS server. (A) prM and DC-SIGN docking complex. (B) Main-chain deformability. (C) B-factor values. (D) The eigenvalue. (E) Variance. (F) Co-variance map. (G) Elastic network of model.

Supplementary Figure S8: *Normal Mode Analysis of Selected vaccine proteins and Dengue immune receptors.*

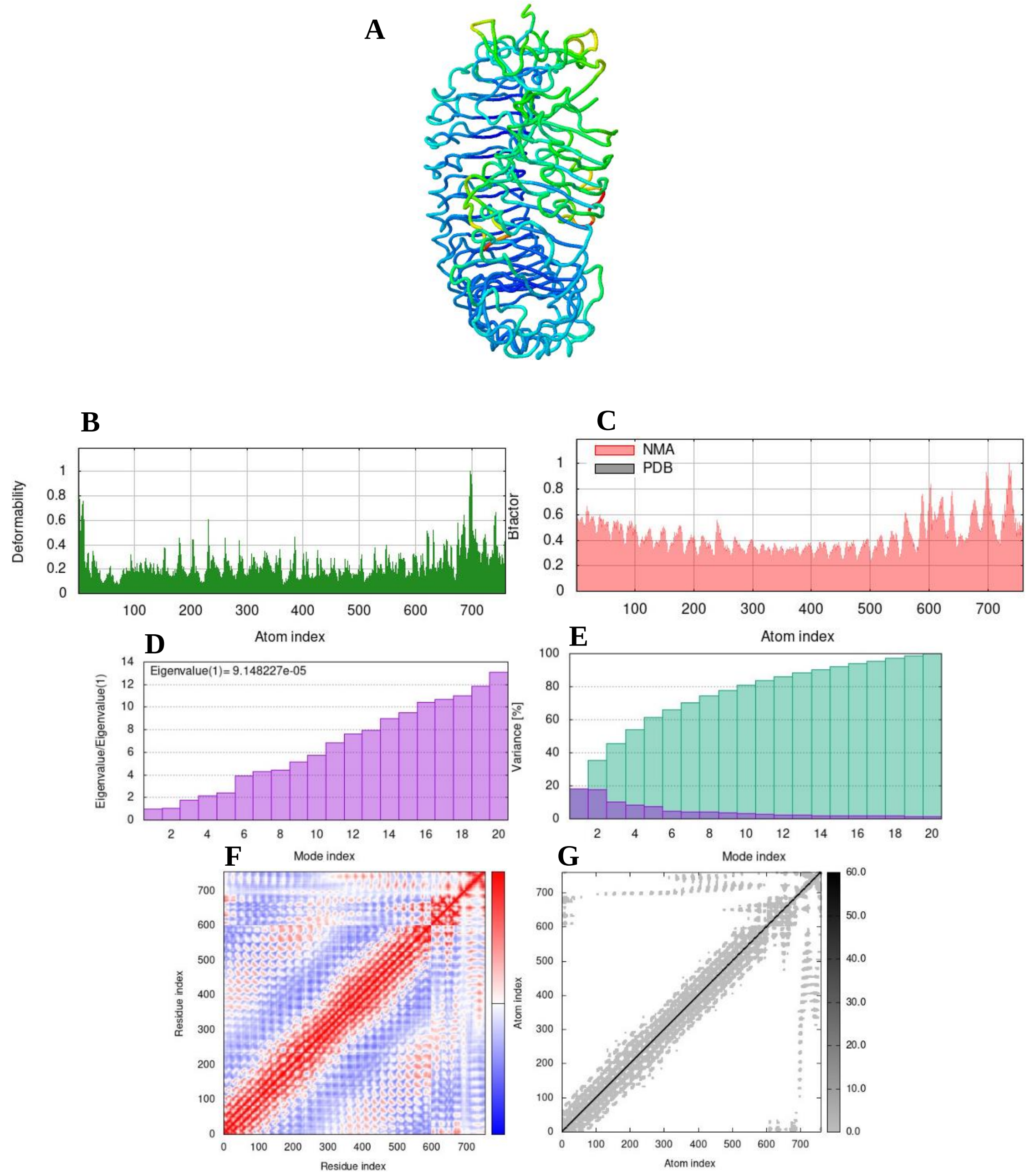

Figure S8 (vi): Molecular dynamics simulation of prM and TLR4 docking complex by iMODS server. (A) prM and TLR4 docking complex. (B) Main-chain deformability. (C) B-factor values. (D) The eigenvalue. (E) Variance. (F) Co-variance map. (G) Elastic network of model.

Supplementary Figure S8: *Normal Mode Analysis of Selected vaccine proteins and Dengue immune receptors.*

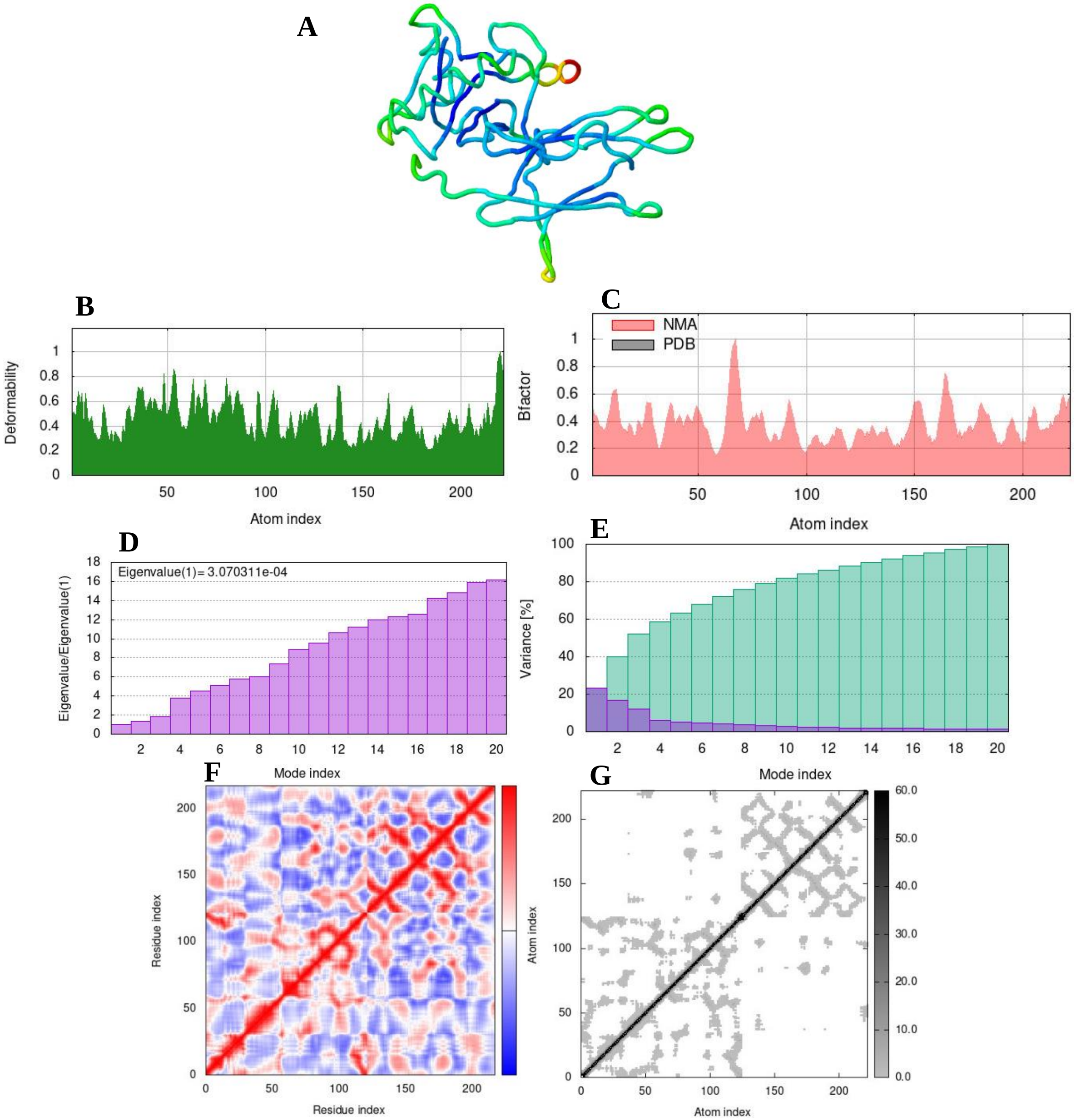

Figure S8 (vii): Molecular dynamics simulation of EIII and MR docking complex by iMODS server. (A) EIII and MR docking complex. (B) Main-chain deformability. (C) B-factor values. (D) The eigenvalue. (E) Variance. (F) Co-variance map. (G) Elastic network of model.

Supplementary Figure S8: *Normal Mode Analysis of Selected vaccine proteins and Dengue immune receptors.*

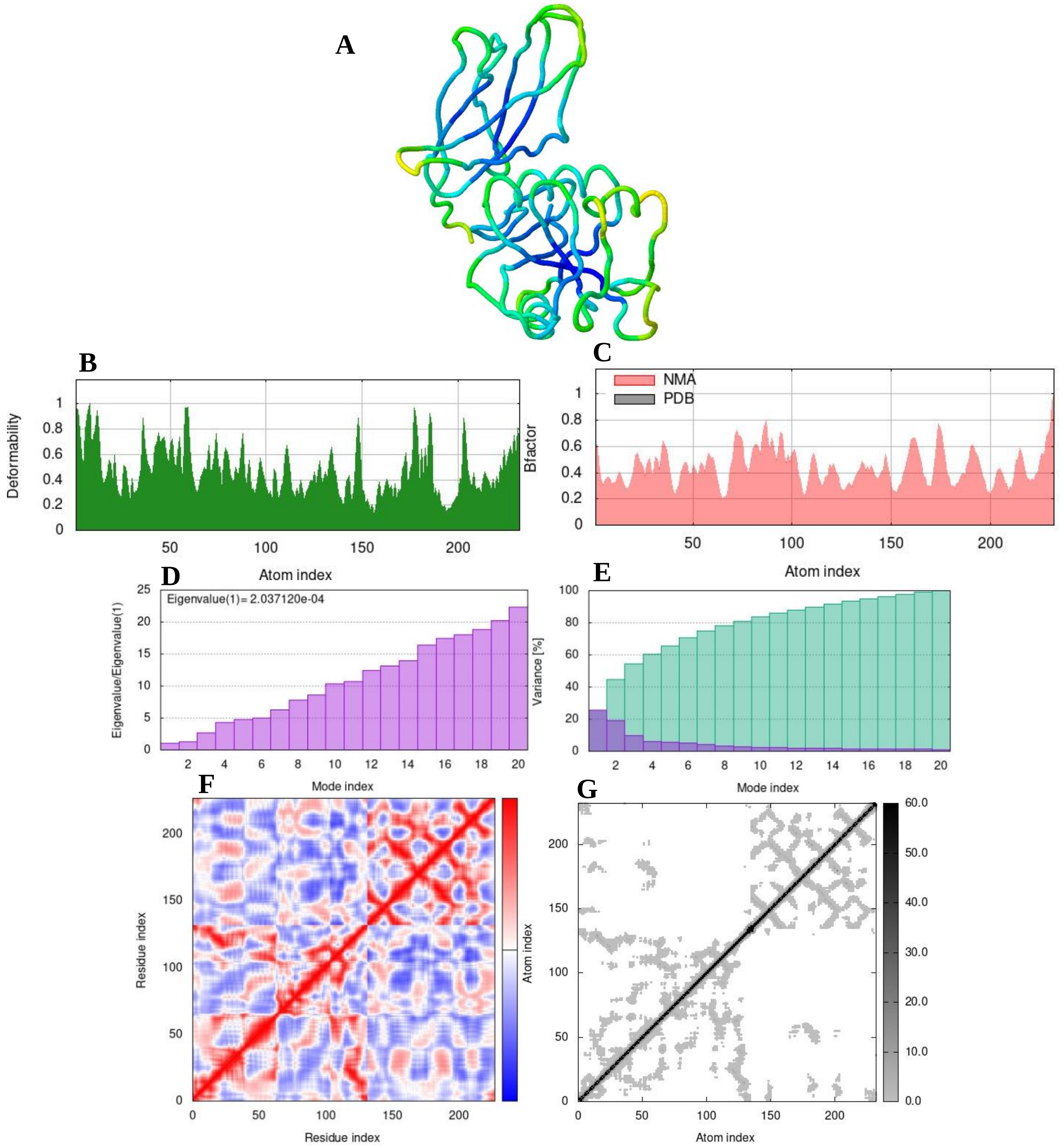

Figure S8 (viii): Molecular dynamics simulation of EIII and DC-SIGN docking complex by iMODS server. (A) EIII and DC-SIGN docking complex. (B) Main-chain deformability. (C) B-factor values. (D) The eigenvalue. (E) Variance. (F) Co-variance map. (G) Elastic network of model.

Supplementary Figure S8: *Normal Mode Analysis of Selected vaccine proteins and Dengue immune receptors.*

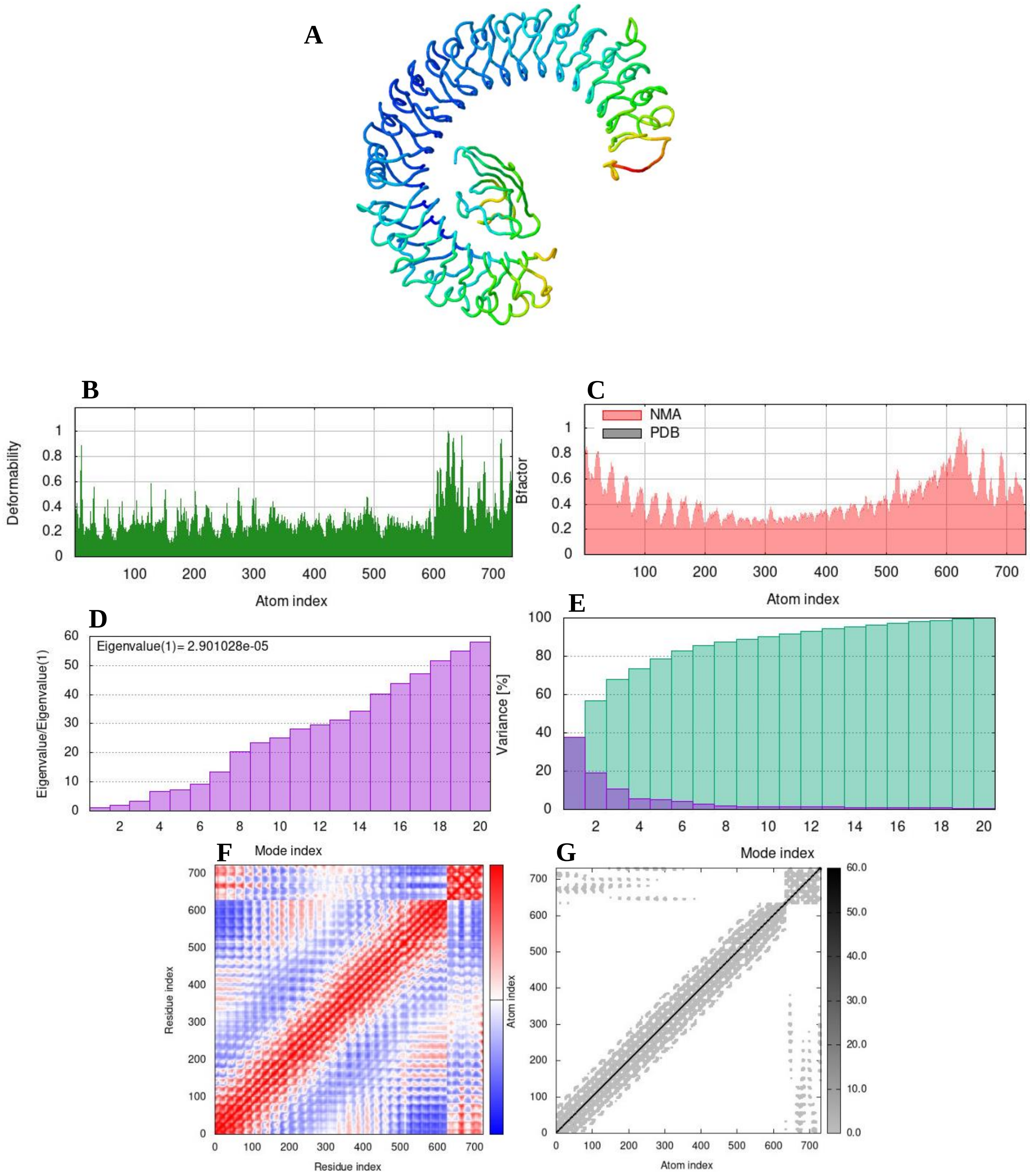

Figure S8 (ix): Molecular dynamics simulation of EIII and TLR3 docking complex by iMODS server. (A) EIII and TLR3 docking complex. (B) Main-chain deformability. (C) B-factor values. (D) The eigenvalue. (E) Variance. (F) Co-variance map. (G) Elastic network of model.
