# Supplementary material for: Engineering Modified mRNA-Based Vaccine against Dengue Virus Using Computational and Reverse Vaccinology Approaches
Source: Int J Mol Sci. 2022 Nov 11;23(22):13911. doi: 10.3390/ijms232213911 (PMC9698390; doi:10.3390/ijms232213911)
Supplement: Supplementary file 1 [file ijms-23-13911-s001.zip › Table S1.pdf]

Supplementary Table S1: *Physicochemical Properties of the Conserved Protein*

Table S1. Physicochemical Properties of the Conserved Protein

Parameters	Proteins		
	**NS1**	**prM**	**E-III**
**Antigenicity**	0.6202	0.5479	0.6526
**Allergenicity**	Probable Allergen	Probable non-Allergen	Probable non-Allergen
**Toxicity**	Non toxin	Non toxin	Non toxin
**Theoretical Isoelectric Point (pI)**	6.82	5.79	8.03
**Extinction coefficient**	84170	33835	15470
**Instability Index**	41.66	47.16	38.38
**Aliphatic Index**	69.41	82.45	97.29
**Molecular Weight**	40011.54	17498.25	10980.81
**Number of amino acids**	355	155	97
**Grand average of hydropathicity (GRAVY)**	-0.521	-0.052	-0.264
