# Supplementary material for: Engineering Modified mRNA-Based Vaccine against Dengue Virus Using Computational and Reverse Vaccinology Approaches
Source: Int J Mol Sci. 2022 Nov 11;23(22):13911. doi: 10.3390/ijms232213911 (PMC9698390; doi:10.3390/ijms232213911)
Supplement: Supplementary file 1 [file ijms-23-13911-s001.zip › Table S2.pdf]

Supplementary Table S2. *The nucleotide sequence from the N-terminal to C-terminal direction of the mRNA vaccine (NS1, prM and EIII).*

**NS1**	5′m7GpppmACAUUUGCUUCUGACACAACUGUGUUCACUAGCAACCUCAAACAGACACCGCCGCCAUGAUGGACGCCAUGAAGAGGGGCCUGUGCUGCGUGCUGCUGCUGUGCGGCGCCGUGUUCGUGAGCCCCAGCGGCUGCGUGGUGAGCUGGAAGAAGGAGCUGAAGUGCGGCGGCAUCUUCUUCACCGACAACGUGCACACCACCACCGAGCAGUACAAGUUCCAGCCCGAGAGCCCCAGCAAGCUGGCCAGCGCCAUCCAGAAGGCCCACGAGGAGGGCAUCUGCGGCAUCCGCAGCGUGACCCGCCUGGAGAACCUGAUGUGGAAGCAGAUCACCCCCGAGCUGAACCACAUCCUGAGCGAGAACGAGGUGAAGCUGAGCAUCAUGACCGGCGACAUCAAGGGCAUCAUGCAGGCCGGCAAGCGCAGCCUGCGCCCCCAGCCCACCGAGCUGAAGUACAGCUGGAAGACCUGGGGCAAGGCCAAGAUGCUGAGCACCGAGCCCCACAACCAGACCUUCCUGAUCGACGGCCCCGAGACCGCCGAGUGCCCCAACACCAACCGCGCCUGGAACAGCCUGGAGGUGGAGGACUACGGCUUCGGCGUGUUCACCACCAACAUCUGGCUGAAGCUGAAGGAGCGCCAGGACGUGUUCUGCGACAGCAAGCUGAUGAGCGCCGCCAUCAAGGACAACCGCGCCGUGCACGCCGACAUGGGCUACUGGAUCGAGAGCGCCCUGAACGACACCUGGAAGAUCGAGAAGGCCAGCUUCAUCGAGGUGAAGAGCUGCCACUGGCCCAAGAGCCACACCCUGUGGAGCAACGGCGUGCUGGAGAGCGAGAUGAUCAUCCCCAAGAACUUCGCCGGCCCCGUGAGCCAGCACAACUACCGCCCCGGCUACCACACCCAGACCGCCGGCCCCUGGCACCUGGGCAAGCUGGAGAUGGACUUCGACUUCUGCGAGGGCACCACCGUGGUGGUGACCGAGGACUGCGGCAACCGCGGCCCCAGCCUGCGCACCACCACCGCCAGCGGCAAGCUGAUCACCGAGUGGUGCUGCCGCAGCUGCACCCUGCCCCCCCUGCGCUACCGCGGCGAGGACGGCUGCUGGUACGGCAUGGAGAUCCGCCCCCUGAAGGAGAAGGAGGAGAACCUGGUGAACAGCCUGGUGACCGCCGGCCACGGCGCUCGCUUUCUUGCUGUCCAAUUUCUAUUAAAGGUUCCUUUGUUCCGUAAGUCCAACUACUAAACUGGGGGAUAUUAUGAAGGGCCUUGAGCAUCUGGAUUCUGCCUAAUAAAAAACAUUUAUUUUCAUUGCGCUCGCUUUCUUGCUGUCCAAUUUCUAUUAAAGGUUCCUUUGUUCCGUAAGUCCAACUACUAAACUGGGGGAUAUUAUGAAGGGCCUUGAGCAUCUGGAUUCUGCCUAAUAAAAAACAUUUAUUUUCAUUGCAAAAAAAAAAAAAAAAAAAAAAAAAAAAAAAAAAAAAAAAAAAAAAAAAAAAAAAAAAAAAAAAAAAAAAAAAAAAAAAAAAAAAAAAAAAAAAAAAAAAAAAAAAAAAAAAAAAAAAAAAAAAAAAAAAA3′
**prM**	5’m7GpppmACAUUUGCUUCUGACACAACUGUGUUCACUAGCAACCUCAAACAGACACCGCCGCCAUGAUGGACGCCAUGAAGAGGGGCCUGUGCUGCGUGCUGCUGCUGUGCGGCGCCGUGUUCGUGAGCCCCAUGAUCGUGAGCAAGCAGGAGAAGGGCAAGAGCCUGCUGUUCAAGACCGAGGACGGCGUGAACAUGUGCACCCUGAUGGCCAUGGACCUGGGCGAGCUGUGCGAGGACACCAUCACCUACAACUGCCCCCUGCUGCGCCAGAACGAGCCCGAGGACAUCGACUGCUGGUGCAACGCCACCAGCACCUGGGUGACCUACGGCACCUGCACCGCCACCGGCGAGCACCGCCGCGAGAAGCGCAGCGUGGCCCUGGUGCCCCACGUGGGCAUGGGCCUGGAGACCCGCACCGAGACCUGGAUGAGCAGCGAGGGCGCCUGGAAGCACGCCCAGCGCAUCGAGACCUGGAUCCUGCGCCACCCCGGCUUCACCAUCAUGGCCGCCAUCCUGGCCUACACCAUCGGCACCACCUACUUCCAGCGCGUGCUGAUCUUCAUCCUGCUGACCGCCGUGGCCCCCAGCAUGACCGCUCGCUUUCUUGCUGUCCAAUUUCUAUUAAAGGUUCCUUUGUUCCGUAAGUCCAACUACUAAACUGGGGGAUAUUAUGAAGGGCCUUGAGCAUCUGGAUUCUGCCUAAUAAAAAACAUUUAUUUUCAUUGCGCUCGCUUUCUUGCUGUCCAAUUUCUAUUAAAGGUUCCUUUGUUCCGUAAGUCCAACUACUAAACUGGGGGAUAUUAUGAAGGGCCUUGAGCAUCUGGAUUCUGCCUAAUAAAAAACAUUUAUUUUCAUUGCAAAAAAAAAAAAAAAAAAAAAAAAAAAAAAAAAAAAAAAAAAAAAAAAAAAAAAAAAAAAAAAAAAAAAAAAAAAAAAAAAAAAAAAAAAAAAAAAAAAAAAAAAAAAAAAAAAAAAAAAA3’
**EIII**	5’m7GpppmACAUUUGCUUCUGACACAACUGUGUUCACUAGCAACCUCAAACAGACACCGCCGCCAUGAUGGACGCCAUGAAGAGGGGCCUGUGCUGCGUGCUGCUGCUGUGCGGCGCCGUGUUCGUGAGCCCCAGCUACGCCAUGUGCACCAACAAGUUCAAGGUGGUGAAGGAGAUCGCCGAGACCCAGCACGGCACCAUCGUGGUGCGCGUGCAGUACGAGGGCGACGGCAGCCCCUGCAAGAUCCCCUUCGAGAUCAUGGACCUGGAGAAGCGCCACGUGCUGGGCCGCCUGAUCACCGUGAACCCCAUCGUGACCGAGAAGGACAGCCCCGUGAACAUCGAGGCCGAGCCCCCCUUCGGCGACAGCUACAUCAUCAUCGGCGUGGAGCCCGGCCAGCUGAAGCUGAGCUGGUUCAAGAAGGCUCGCUUUCUUGCUGUCCAAUUUCUAUUAAAGGUUCCUUUGUUCCGUAAGUCCAACUACUAAACUGGGGGAUAUUAUGAAGGGCCUUGAGCAUCUGGAUUCUGCCUAAUAAAAAACAUUUAUUUUCAUUGCGCUCGCUUUCUUGCUGUCCAAUUUCUAUUAAAGGUUCCUUUGUUCCGUAAGUCCAACUACUAAACUGGGGGAUAUUAUGAAGGGCCUUGAGCAUCUGGAUUCUGCCUAAUAAAAAACAUUUAUUUUCAUUGCAAAAAAAAAAAAAAAAAAAAAAAAAAAAAAAAAAAAAAAAAAAAAAAAAAAAAAAAAAAAAAAAAAAAAAAAAAAAAAAAAAAAAAAAAAAAAAAAAAAAAAAAAAAAAAAAAAAAAAAAAA3’
